# Advancing Gel Systems with Natural Extracts: Antioxidant, Antimicrobial Applications, and Sustainable Innovations

**DOI:** 10.3390/gels11020125

**Published:** 2025-02-08

**Authors:** Arthitaya Kawee-ai

**Affiliations:** Division of Cannabis and Medicinal Plants for Local Development, Graduate School, Payap University, Chiang Mai 50000, Thailand; kaweeai@gmail.com

**Keywords:** natural extracts, gel systems, antioxidant properties, antimicrobial activity, bioactive compounds, nanotechnology

## Abstract

The integration of natural extracts into gel systems has emerged as a transformative approach to enhance functional properties, including antioxidant, antimicrobial, and therapeutic effects. This review underscores the remarkable potential of natural extract-enriched gels, which effectively combine sustainability with improved functionality. These bioactive compounds, sourced from plants and animals, encompass polyphenols, flavonoids, essential oils, chitosan, proteins, and polysaccharides. They provide an eco-friendly alternative to synthetic additives and find applications across various sectors, including pharmaceuticals, cosmetics, and food packaging. Despite their promise, challenges remain, such as the variability in natural extract composition, the stability of bioactive compounds, and scalability for industrial use. To address these issues, innovative strategies like nanoencapsulation, responsive hydrogels, and AI-driven optimization have demonstrated significant progress. Additionally, emerging technologies, such as 3D printing and adherence to circular economy principles, further enhance the versatility, efficiency, and sustainability of these systems. By integrating these advanced tools and methodologies, gel systems enriched with natural extracts are well-positioned to meet contemporary consumer and industrial demands for multifunctional and eco-friendly products. These innovations not only improve performance but also align with global sustainability goals, setting the stage for widespread adoption and continued development in various fields.

## 1. Introduction

Gels have emerged as one of the most flexible and adaptable materials in modern science and technology, with uses in a variety of sectors, including medicines, cosmetics, food technology, and biotechnology [1,2]. Gels are semi-solid structures with a three-dimensional network capable of immobilizing a huge volume of liquid [3]. They have unique structural and functional features that make them excellent for delivering bioactive substances in a variety of situations [4,5]. Their customizable rheological features, simplicity of manufacture, and capacity to maintain and regulate the release of active substances have accelerated their usage in the development of novel solutions for both industrial and medicinal applications [3,4]. Over the past few decades, there has been a concerted effort to enhance the functionality of gels to meet the growing demand for bioactive materials. Among the most sought-after functionalities are antioxidant and antimicrobial properties, which hold immense significance across multiple domains. Antioxidants are crucial in mitigating oxidative stress caused by an imbalance between reactive oxygen species (ROS) and the body’s ability to detoxify them [6]. Oxidative stress has been linked to various chronic diseases, including cancer, cardiovascular disorders, and neurodegenerative conditions [7]. Similarly, antimicrobial properties are indispensable for preventing and controlling microbial contamination, which poses significant challenges in healthcare, food preservation, and personal care [8,9].

Traditionally, synthetic antioxidants and antimicrobial agents, such as butylated hydroxytoluene (BHT), butylated hydroxyanisole (BHA), parabens, and triclosan, have been widely employed to impart these functionalities to gels [10,11,12]. However, concerns regarding their potential toxicity, environmental impact, and allergenic effects have driven a shift toward exploring natural alternatives [12,13]. Consumers increasingly favor eco-friendly, sustainable, and naturally derived products, emphasizing the need for bio-based solutions [14]. Natural extracts derived from plants and animals have garnered significant attention as a viable alternative to synthetic additives. Rich in bioactive compounds, such as polyphenols, flavonoids, alkaloids, terpenes, and peptides, these extracts offer potent antioxidant and antimicrobial properties [15]. For example, green tea, turmeric, and essential oils have shown remarkable success in neutralizing free radicals and inhibiting microbial growth [16,17,18,19]. Similarly, animal-derived substances, such as chitosan and honey, exhibit antimicrobial and wound-healing properties, making them valuable additions to gel systems [20,21].

Integrating natural extracts into gel systems poses both opportunities and challenges. On the one hand, these bioactive compounds have the potential to significantly enhance the functional properties of gels, making them more effective in various applications. For instance, the incorporation of antioxidants can improve the stability of gels against oxidative degradation, while antimicrobial agents can extend the shelf life of products and reduce the risk of microbial contamination [22,23]. However, the successful integration of natural extracts into gels requires addressing several technical challenges, such as ensuring the stability of bioactive compounds during processing and storage, achieving controlled release for sustained functionality, and maintaining compatibility with the gel matrix [24,25,26]. This review delves into the role of natural extracts in enhancing the properties of gel systems, with a particular focus on their antioxidant and antimicrobial applications. Analyzing the interactions between natural extracts and gel matrices underscores recent advancements, addresses existing challenges, and outlines directions for future research. This comprehensive understanding sets the stage for the development of innovative and sustainable solutions across modern scientific and industrial fields.

## 2. Natural Extracts Sources and Properties

**Figure 1:**
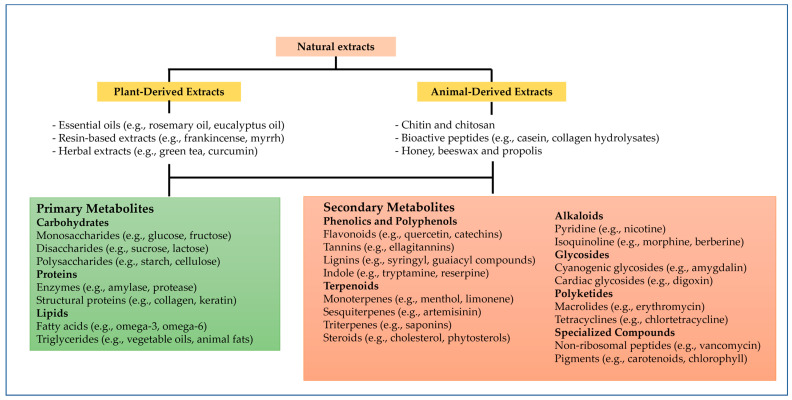
Natural extracts sources and possible bioactive compounds.

Natural extracts have been extensively studied for their multifunctional properties, which make them valuable for enhancing the antioxidant and antimicrobial capabilities of gel systems. These extracts are derived from a wide range of natural sources, including plants and animals, and are rich in bioactive compounds, such as polyphenols, alkaloids, terpenes, and peptides [15], as presented in Figure 1. Their efficacy in diverse applications is closely linked to their origin, chemical composition, and the mechanisms underlying their bioactivity.

Plants are among the most widely studied sources of natural bioactive compounds. They produce secondary metabolites, such as polyphenols, flavonoids, tannins, and terpenes, which exhibit strong antioxidant and antimicrobial properties. Plant-derived extracts (Figure 1) include essential oils, resin-based extracts, and herbal extracts containing primary metabolites, such as carbohydrates, proteins, and lipids (e.g., glucose, collagen, omega-3 fatty acids). Secondary metabolites, prominent in these extracts, comprise phenolics (e.g., flavonoids, tannins), terpenoids (e.g., menthol, steroids), alkaloids (e.g., nicotine, berberine), glycosides (e.g., amygdalin, digoxin), polyketides (e.g., erythromycin), and specialized compounds like pigments (e.g., chlorophyll) [15]. For example, green tea (*Camellia sinensis*) is rich in catechins, a type of polyphenol known for its free radical-scavenging ability [27,28]. Similarly, curcumin from turmeric (*Curcuma longa*) has demonstrated potent antimicrobial activity against a broad spectrum of bacteria, including multidrug-resistant strains [29,30]. Essential oils derived from plants, such as oregano (*Origanum vulgare*), thyme (*Thymus vulgaris*), and eucalyptus (*Eucalyptus globulus*), are particularly effective due to their high concentration of bioactive terpenes and phenolic compounds [31].

Animal-derived extracts include components such as chitin, bioactive peptides, and products like honey and beeswax (Figure 1). These extracts provide bioactive compounds and structural proteins, playing a pivotal role in biotechnological, medicinal, and industrial applications [20,32,33]. Additionally, animal-derived substances, such as chitosan, honey, and propolis, exhibit significant bioactivity. Chitosan, a polysaccharide obtained from the exoskeletons of crustaceans, has antimicrobial properties that stem from its ability to disrupt microbial cell membranes and inhibit DNA replication [34]. Honey, a natural product of bees, combines antioxidant activity due to its phenolic content with antimicrobial effects mediated by its osmotic properties and the production of hydrogen peroxide [33,35]. Propolis, another bee product, is rich in flavonoids and phenolic acids, making it an effective antimicrobial agent for use in gels [33].

## 3. Gel Matrices and Interaction with Natural Extracts

The structural and functional properties of gel systems play a significant role in determining their suitability for various applications. The incorporation of natural extracts into these matrices requires a deep understanding of the types of gel systems, their physicochemical properties, and the interactions that occur between the gel components and bioactive extracts [25,36].

### 3.1. Types of Gel Matrices

Gels can be classified based on the polarity of their liquid phase: hydrogels contain a polar liquid phase, such as water, while organogels are characterized by an apolar liquid phase, such as oil [5,37].

Hydrogels are among the most commonly used gel systems for incorporating natural extracts due to their high-water content and biocompatibility [5,37]. These systems are composed of hydrophilic polymers, such as alginate, agarose, carrageenan, or polyvinyl alcohol, which form a three-dimensional network capable of retaining water or aqueous solutions [38]. The hydrophilic nature of hydrogels makes them ideal for delivering water-soluble bioactive compounds like polyphenols and flavonoids. For example, alginate-based hydrogels have been used to encapsulate phenolic compounds from green tea, enhancing their stability and controlled release [39,40].

Organogels are non-aqueous gel systems formed by structuring organic liquids, such as oils, using gelators like lecithin, sorbitan monostearate, or waxes, while organogels composed of edible compounds are termed “oleogels” [37]. Organogels gels are particularly suitable for incorporating lipophilic natural extracts, such as essential oils and carotenoids, which may have limited solubility in aqueous systems [41,42]. For instance, thyme essential oil has been successfully integrated into a chitosan–benzoic acid-based nanogel for antimicrobial applications in food preservation [43].

Hybrid gels combine the properties of hydrogels and organogels to achieve multifunctionality [37]. These systems can incorporate both hydrophilic and lipophilic natural extracts, broadening their applicability. Hybrid gels have been explored in cosmetic formulations to deliver combinations of water-soluble antioxidants and lipid-soluble vitamins, achieving synergistic effects [44].

### 3.2. Role of Natural Extracts in Gelation Mechanisms

**Figure 2:**
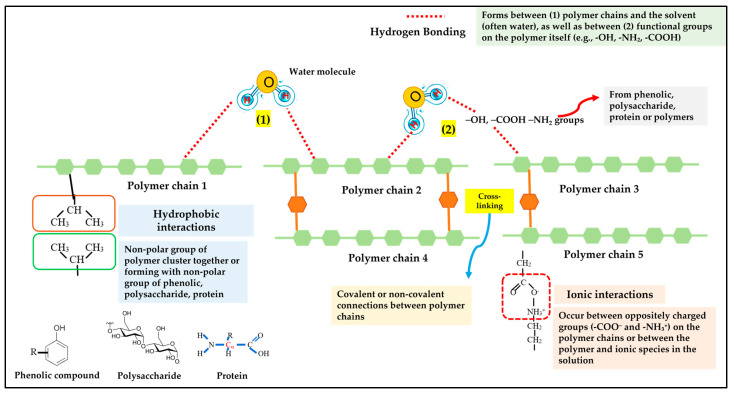
Possible mechanisms of phenolic compounds, polysaccharides, and proteins in gelation.

Plant extracts, rich in bioactive compounds, such as phenolics, polysaccharides, and proteins, contribute to the gelation process through a combination of chemical and physical interactions [36,42,45,46,47], as presented in Figure 2.

#### 3.2.1. Phenolic Compounds

Phenolic compounds are recognized for their antioxidant properties and their ability to form strong interactions with other molecules [48]. Their role in gelation includes the following mechanisms:

1. Hydrogen bonding: phenolic hydroxyl (–OH) groups form hydrogen bonds with water molecules and other gel matrix components, enhancing the stability of the network [49].
2. Hydrophobic interactions: the aromatic rings in phenolic compounds create nonpolar interactions, contributing to the gel’s structural integrity [50,51].
3. Covalent cross-linking: under oxidative conditions, phenolics can form covalent bonds with proteins or other molecules, resulting in a tightly cross-linked gel network [52].

#### 3.2.2. Polysaccharides

Polysaccharides are long-chain carbohydrates that exhibit excellent gel-forming capabilities due to their structural and chemical properties [53,54]. Their contribution to gelation involves the following:

1. Hydrogen bonding: hydroxyl groups on polysaccharides facilitate extensive hydrogen bonding, which is crucial for network formation in gels [54].
2. Ionic interactions: polysaccharides with charged functional groups (e.g., carboxyl or sulfate groups) interact electrostatically with oppositely charged molecules, aiding in cross-linking and gel stabilization [54].
3. Physical entanglement: polysaccharide chains interweave, forming a three-dimensional network that traps water and other compounds, creating the gel’s structure.

#### 3.2.3. Proteins

Proteins in plant extracts contribute to gelation by undergoing structural changes and forming new bonds [53,55]. This occurs through the following:

1. Denaturation and Aggregation: proteins may unfold under specific conditions (e.g., changes in temperature or pH), exposing reactive sites that facilitate bonding [55].
2. Cross-linking: reactive groups in proteins (e.g., amino, carboxyl, and sulfhydryl groups) form covalent bonds, stabilizing the gel network [53,56].
3. Hydrophobic interactions: nonpolar regions of proteins interact with other hydrophobic compounds, reinforcing the gel matrix [55].

#### 3.2.4. Interactions Between Components

The synergistic interaction of phenolics, polysaccharides, and proteins enhances gelation, for example:

1. Phenolics and Proteins: phenolic compounds interact with proteins through hydrogen bonding and covalent linkages, strengthening the gel network [51].
2. Polysaccharides and Proteins: polysaccharides bind to proteins, creating hybrid gels with superior mechanical and functional properties [57].

Once these interactions occur, a three-dimensional gel network is formed. This network traps water and other solutes, giving the gel its characteristic properties, such as elasticity, viscosity, and stability [51,57]. Through these mechanisms, plant extracts significantly contribute to the gelation process, making them valuable in applications requiring tailored gel properties [58]. However, the gelation process is influenced by environmental factors that affect the interactions of these bioactive compounds [55,58]. Changes in pH can alter the ionization state of functional groups, promoting ionic interactions or triggering protein denaturation [59]. Heat can denature proteins and activate certain polysaccharides, initiating the gelation process [58]. Additionally, enzymes can catalyze specific reactions, such as cross-linking, further enhancing gel formation [47,60].

### 3.3. Functional Implications of Interaction in Antioxidant and Antimicrobial Activities

The integration of plant extracts into gel matrices has gained significant attention due to their multifunctional properties and sustainability. Plant extracts, rich in bioactive compounds, such as phenolics, polysaccharides, and proteins, contribute to the gelation process through various mechanisms [36,44,45,59]. A search conducted from 2022 to 2024 using PubMed, Scopus, ScienceDirect, and Google Scholar databases revealed that incorporating natural extracts into various gel-based formulations resulted in notable differences in antioxidant and antimicrobial activities, as shown in Table 1 and Table 2, respectively.

#### 3.3.1. Antioxidant Activities

Plant-based formulations have shown remarkable potential in scavenging free radicals, as evidenced by increased DPPH and ABTS inhibition rates, as presented in Table 1. For example, *Kunzea ericoides* leaf extract hydrogel enhanced ABTS inhibition to 35.35% and DPPH activity to 28.43% [61]. Similarly, encapsulated *Moringa oleifera* seed extract achieved 42.34% DPPH inhibition and 36.78% ABTS activity, highlighting the efficacy of encapsulation techniques in preserving antioxidant compounds [63]. Another significant finding is the use of alginate films with lemon balm and chokeberry pomace wastes. The films with LB displayed superior antioxidant activity, with QUENCHERDPPH values of 469.0–1174.9 μmol TE/100 g, demonstrating their efficacy as sustainable antioxidant materials [65]. In contrast, oleogels incorporating egg white protein and apple polyphenol were effective in inhibiting the generation of oxidation products, focusing more on oxidative stability rather than direct inhibition percentages [68]. Edible films incorporating olive leaf extract also exhibited concentration-dependent increases in DPPH scavenging activity [62,82]. Similarly, oleogels containing tea polyphenols and apple extracts significantly improved oxidative stability and radical scavenging activities, underlining the value of polyphenolic compounds in combating oxidative stress [72]. These sources contribute diverse bioactive compounds, primarily polyphenols, flavonoids, essential oils, and terpenoids, which exhibit significant antioxidant activity through multiple mechanisms [61,62,67].

As presented in Table 1, natural compounds with a higher number of hydroxyl groups often exhibit significant antioxidant activity due to the strong hydrogen-donating ability of these groups [61,63,67,68]. Additionally, the arrangement of hydroxyl groups can alter the molecular electrostatic potential of phenolic compounds, thereby influencing their reactivity and functionality [48]. Natural extracts, rich in bioactive compounds, such as phenolics, flavonoids, and carotenoids, possess strong antioxidant properties [81,82]. These compounds combat oxidative stress and enhance gel stability through the following mechanisms:

1. Inhibition of Oxidation-Induced Degradation

Reactive oxygen species (ROS) and free radicals can degrade gel matrices over time, affecting their texture, elasticity, and shelf life [68,69]. Antioxidants neutralize these species, preventing oxidative damage [77,78]. As a result, the gel maintains its structural integrity, ensuring consistent performance in applications such as cosmetics and wound dressings [61,67,81].

2. Protection of Sensitive Ingredients

Gels often incorporate sensitive ingredients, such as vitamins, enzymes, or active pharmaceutical compounds [69,70,75,79]. Antioxidants in natural extracts protect these ingredients from oxidative degradation, thereby prolonging the efficacy and shelf life of gels containing bioactive agents [17,74].

3. Improved Mechanical Properties

Antioxidants can interact with gel-forming agents, reinforcing the network through secondary interactions (e.g., hydrogen bonding). This enhances the gel’s elasticity, firmness, and resistance to mechanical stress [48,49].

These findings have significant implications for the development of functional materials in food preservation, packaging, and healthcare applications [8,61,67,69,70,75,79,81]. The successful integration of waste products, such as lemon balm and chokeberry pomace, into effective antioxidant materials, also suggests promising directions for sustainable material development [65]. Future research should focus on optimizing delivery systems and investigating the long-term stability of these antioxidant properties under various environmental conditions.

#### 3.3.2. Antimicrobial Activities

The antimicrobial efficacy of the formulations varied across different gel types (Table 2). In addition to antioxidant benefits, many plant-based gels and films demonstrated broad-spectrum antimicrobial activity. Hydrogels incorporating *Calendula officinalis* extract exhibited intermediate antimicrobial effects against pathogens, including *Staphylococcus aureus, Escherichia coli*, and *Pseudomonas aeruginosa* [81]. Likewise, *Rosmarinus officinalis* nanoemulgel showed potent efficacy, with minimum inhibitory concentrations (MICs) of 2.3, 3.75, and 6.5 µg/mL against *Pseudomonas aeruginosa, Klebsiella pneumoniae*, and methicillin-resistant Staphylococcus aureus, respectively [19]. Hydrogels containing *Eucalyptus Camaldulensis* were active against *Acinetobacter baumannii* and *Staphylococcus epidermidis* [84], while those with ovalbumin amyloid fibrils (combined with tannic acid or EGCG) exhibited broad-spectrum antibacterial activity against both Gram-positive and Gram-negative bacteria [69,70]. Nanoemulsions with *Grumixama* leaves effectively inhibited the growth of foodborne pathogens, including *Staphylococcus aureus, Bacillus cereus*, and *Listeria monocytogenes* [85]. Phytosomal gels of *Annona squamosa* and *Cinnamomum tamala* leaves reduced bacterial load in animal models, demonstrating potential in clinical applications [86].

**Table 2:** The interactions of natural extracts with various gel-based formulations as antimicrobial activity.

Extracted Sources	Active Compounds	Gel Forms	Effect of Adding a Natural Extract	References
*Curcuma longa* L.	Curcuminoids	Hydrogel	The gel with curcumin encapsulated at 350 and 700 µg/mL exhibited antibacterial efficacy against *Staphylococcus aureus* and *Escherichia coli*, while the gel without curcumin at 700 µg/mL also showed antimicrobial activity.	[16]
*Syzygium aromaticum* essential oil	Eugenol, terpenes and terpenoids, sesquiterpenes, phenolic compounds, and aldehydes	Hydrogel	Essential oil loading into hydrogels enhances the antimicrobial activity against *S. aureus* by 51%, *E. coli* by 45%, and Candida albicans by 21%.	[18]
Rosemary (*Rosmarinus officinalis*) essential oil	Flavone compounds, diterpenes, steroidal derivatives, and triterpenes	Nanoemulgel	The nanoemulgel demonstrated activity against *Pseudomonas aeruginosa, Klebsiella pneumoniae, and Methicillin-resistant S. aureus*, with minimum inhibitory concentrations (MICs) of 2.3, 3.75, and 6.5 µg/mL, respectively.	[19]
*Oliveria decumbens* Vent. essential oil	Phenolics, essential oils	Gelatin-based films	The incorporation of *Oliveria decumbens* Vent. essential oil into the film resulted in inhibition zones of 33.75 mm against *E. coli* and 38.21 mm against *S. aureus*.	[66]
Tragacanth gum and clove	Phenolics, polysaccharides, eugenol, gallic acid, flavonoids, and anthocyanins	Hydrogel	The hydrogel containing tragacanth gum and 3% or 5% clove extract exhibited inhibitory activity against *E. coli, S. aureus*, and *Salmonella enterica*, with a clear inhibition zone greater than 15 mm. However, it showed no activity against *Listeria monocytogenes, Bacillus subtilis*, or *Yersinia enterocolitica*.	[67]
Ovalbumin amyloid fibrils + tannic acid	Phenolics, protein	Hydrogel	The hydrogel containing ovalbumin amyloid fibrils and 2% tannic acid reduced the growth of *E. coli* from 7.77 log CFU/mL to 4.86 log CFU/mL and *S. aureus* from 7.62 log CFU/mL to 4.70 log CFU/mL.	[69]
Ovalbumin amyloid fibrils + epigallocatechin gallate (EGCG)	Polyphenols, protein	Hydrogel	The EGCG-binding ovalbumin amyloid fibrils hydrogel treatment reduced the concentration of *E. coli* from 7.8 to 4.7 log CFU/mL and *S. aureus* from 7.7 to 4.2 log CFU/mL.	[70]
*Aloe vera* gel	Flavonoids, phenolics	Chitosan nanoparticles	*Aloe vera* gel incorporated with nanoparticles exhibited the highest inhibition zones of 28 mm and 30 mm against resistant and sensitive strains of *Helicobacter pylori*, respectively. The MIC was 3.9 µg/mL, while the minimal bactericidal concentration (MBC) was 7.8 µg/mL.	[71]
Rosemary extract	Phenolic compounds	Hydrogel, Oleogel	The application of hydrogel and oleogel with rosemary extract as a coating for sardine fillets delayed the growth of *Enterobacteriaceae* and *Pseudomonas* spp. compared to the untreated samples. The bacterial count of *Enterobacteriaceae* decreased from 6.96 to 5.00 log CFU/mL, while *Pseudomonas* spp. decreased from 8.28 to 7.43 log CFU/mL after 5 days of storage.	[77]
Soybean lipophilic protein + Thyme essential oils + glycerol monolaurate (GML)	Phenolic compounds, protein	Oleogel	Oleogel containing 1–2% GML exhibited a stronger inhibitory effect against *E. coli* and *S. aureus* compared to 3–4% GML. The addition of thymol essential oil enhanced the antibacterial effect, showing greater activity against Gram-positive bacteria than Gram-negative bacteria.	[79]
*Calendula officinalis* extract	Phenolic acids and flavonoid compounds	Hydrogel	The incorporation of hydrogel with 3.9 mg of *Calendula officinalis* extract resulted in an inhibition zone of 12 mm against *S. aureus* and 11.6 mm against *E. coli*.	[81]
Olive leaf	Phenolic compounds	Edible film	The films with olive leaf extracts are effective in inhibiting the growth of all tested bacteria in a dose-dependent manner, in both Gram-positive and Gram-negative strains.	[82]
Keratin from chicken feathers	Keratin and citric acid	Film	The film combined with keratin and citric acid inhibited the growth of E. coli and S. aureus 2 to 3 times more effectively than the film or keratin alone.	[83]
*Eucalyptus Camaldulensis*	kaempferol, corchoionoside B, quinic acid, and quercetin, gallic acid, ellagic acid, phenolics and flavonoids	Hydrogel	Hydrogel containing the hydrophobic fraction of *Eucalyptus camaldulensis* extract demonstrated MIC/MBC values of 78.12/312.50 µg/mL against *Acinetobacter baumannii* and *Staphylococcus epidermidis*.	[84]
*Grumixama* leaves + grape seed oil	Phenolics, flavonoids	Nanoemulsion	A nanoemulsion containing 200 mg of *Grumixama* leaf extract and 100 mg of grape seed oil demonstrated inhibitory effects against *S. aureus, Bacillus cereus*, and *Listeria monocytogenes*, with an inhibition zone of 7.2–7.5 mm. It also exhibited activity against *Salmonella Typhimurium* and *E. coli*, with an inhibition zone of 5.5 mm.	[85]
*Annona squamosa* and *Cinnamomum tamala* leaves	Phenols, flavonoids, glycosides, saponins, tannins, alkaloids, steroids, essential oils, and carbohydrates	Phytosomal gel (PG)	The phytosomal gel containing the extracts reduced the number of *S. aureus* in treated rats from 1.0 × 10^8^ CFU/g to 4.7 × 10^4^ ± 2.6 × 10^3^ CFU/g for 2% PG and 3.8 × 10^4^ ± 8.8 × 10^2^ CFU/g for 5% PG.	[86]
*Evernia prunastri*	Polysaccharides, protein, phenolic compounds	Bio-based films	The film-containing extracts inhibited the growth of all tested Gram-positive bacterial strains, with the strongest effect against *S. aureus* and *B. cereus* (inhibition zone of 3 mm), followed by *L. monocytogenes* and *S. epidermidis* (2 mm), and *Enterococcus faecalis* (1.5 mm).	[87]
Egyptian propolis extract	Polyphenol compounds, hesperetin, chlorogenic acid, caffeic acid	Hydrogel	Propolis extract-functionalized hydrogels exhibited antimicrobial effects against *E. coli, S. mutans*, and *Candida albicans*, with MIC values of 0.012 mg/mL for *E. coli*, 0.05 mg/mL for *S. mutans*, and 0.025 mg/mL for *C. albicans*.	[88]
*Rosmarinus officinalis* L., *Achillea millefolium* L., *Calendula officinalis* L.	Phenolic compounds	Hydrogel	The hydrogel incorporating *Rosmarinus officinalis* extract exhibited inhibition zones of 10 mm against *S. aureus* and *Pseudomonas aeruginosa*, and 15 mm against *C. albicans*. No antimicrobial activity was observed in the hydrogels incorporating *Achillea millefolium* and *Calendula officinalis* extracts.	[89]
*Azadirachta indica* Oil	Polysaccharides, protein, phenolic compounds	Hydrogel	The nanohydrogel exhibited a significantly higher value of 8.40 log CFU/mL for Gram-negative bacteria *E. coli* compared to 8.34 log CFU/mL for Gram-positive *S. aureus*.	[90]
Honeysuckle leaf	Phenolic compounds	Edible film	The film containing the extract inhibited the growth of test bacteria in both Gram-positive and Gram-negative types, with a particular effectiveness against *S. aureus*.	[91]
Chia seeds (*Salvia hispanica* L.)	Polysaccharides, proteins, phenolic compounds	Edible film	The films exhibited concentration-dependent antimicrobial activities against bacteria, showing higher activity against *S. aureus* compared to *E. coli*, particularly at concentrations exceeding 4%.	[92]
*Origanum vulgare* L. essential oil (OEO)	carvacrol, γ-terpinene and o-cymene	Edible film	Inhibition zones with diameters of 14.7, 22.0, and 22.5 mm were observed on *E. coli* for films containing 0.5%, 1%, and 1.5% OEO, respectively. Similarly, inhibition zones with diameters of 13.9, 17.1, and 19.3 mm were observed on *S. aureus* for films containing 0.5%, 1%, and 1.5% OEO.	[93]

Natural extracts also exhibit strong antimicrobial properties, primarily due to compounds such as alkaloids, tannins, and essential oils [16,19,67,93]. These compounds contribute to maintaining the gel’s safety and functionality through the following mechanisms:

1. Inhibition of Microbial Growth

Antimicrobial agents disrupt microbial cell membranes, inhibit enzyme activity, or interfere with microbial DNA replication [82,92]. This prevents contamination and extends the shelf life of gels, particularly in food and pharmaceutical applications [16,86,88,89,93].

2. Creation of a Self-Sanitizing Surface

The antimicrobial components form an active barrier within the gel matrix, suppressing microbial colonization [84,87]. This ensures hygiene and sterility in applications, such as wound healing gels and cosmetics [16,84,86,88,89,93].

3. Synergistic Effects with Gel Matrices

Certain natural extracts interact strongly with the gel matrix, stabilizing and amplifying antimicrobial effects [67,71,85]. This enables the use of lower concentrations of preservatives, thereby reducing potential side effects or toxicity [18,90].

The versatility of these natural formulations extends beyond their individual properties, as many systems demonstrate dual functionality in both antioxidant and antimicrobial applications [18,19,67,69,70,71,77,79,81,82]. For example, tragacanth gum and clove-based hydrogels exhibited both strong antioxidant activity (>90%) and broad-spectrum antimicrobial effects against both Gram-positive and Gram-negative bacteria [67]. Similarly, olive leaf extract-based edible films showed concentration-dependent increases in both DPPH scavenging activity and antimicrobial efficacy against various pathogens [82]. These findings have significant implications for developing sustainable solutions in food preservation, healthcare applications, and active packaging materials, suggesting a promising direction for replacing synthetic additives with natural alternatives [16,84,86,88,89,93]. However, future research should focus on optimizing these systems for industrial scalability while maintaining their functional properties and investigating their long-term stability under various environmental conditions.

## 4. Challenges and Limitations in the Use of Natural Extracts in Gels

Despite the numerous benefits and applications of natural extracts in gel systems, their incorporation is accompanied by various challenges. These limitations stem from the complex nature of natural extracts, their interactions with gel matrices, and practical considerations in scaling up production and ensuring regulatory compliance [86,94,95,96]. Addressing these challenges is crucial to fully realizing the potential of enhanced gels in diverse applications.

### 4.1. Variability in Composition

Natural extracts derived from plants, animals, or microorganisms exhibit significant variability in their composition due to factors such as the source, extraction method, and environmental conditions [4]. This inherent variability poses challenges to ensuring consistent bioactive properties and performance in gel formulations. For example, the antioxidant activity of phenolic extracts may vary depending on plant species, harvest time, and processing techniques [97,98,99]. Such inconsistencies can undermine the reliability and efficacy of gels formulated with natural extracts, emphasizing the critical need for standardization.

Standardizing the composition of natural extracts is a complex task requiring comprehensive characterization of their chemical profiles and bioactive components. Advanced analytical techniques play a pivotal role in addressing this challenge. High-performance liquid chromatography (HPLC) and mass spectrometry are essential tools for identifying and quantifying the components of natural extracts, ensuring batch-to-batch consistency [18,19,67,84,86]. These techniques enable the precise measurement of key bioactive compounds, thereby reducing variability in gel formulations. Additionally, nuclear magnetic resonance (NMR) spectroscopy and molecular modeling offer significant potential to advance the field. NMR spectroscopy provides detailed structural information on bioactive compounds [100], while molecular modeling elucidates the interactions between these compounds and gel matrices [101,102]. By integrating these insights, researchers can design more effective gel systems that maintain the stability and efficacy of natural extracts.

One of the most promising areas of innovation lies in the development of multifunctional gels capable of addressing multiple challenges simultaneously. Future gels could integrate antioxidant, antimicrobial, and anti-inflammatory properties into a single system, offering comprehensive solutions for applications such as wound healing and food preservation [103,104,105]. For instance, hybrid gels combining natural extracts with nanoparticles, such as silver or zinc oxide, could deliver enhanced antimicrobial efficacy while providing a controlled release of antioxidants [106,107]. In the food sector, multifunctional edible gels may simultaneously enhance flavor, protect bioactive ingredients, and improve food texture [2,3,24]. Similarly, in cosmetics, gels with combined skin-rejuvenating and UV-protective properties could cater to consumer demand for multifunctional products [6,8].

Smart gels that respond to external stimuli, such as temperature, pH, light, or magnetic fields, are emerging as transformative technologies. By incorporating natural extracts into responsive gel matrices, these systems can deliver bioactive compounds in a controlled manner tailored to specific environmental conditions [106]. For example, pH-sensitive hydrogels loaded with plant-derived antioxidants could release their contents in acidic environments, making them ideal for targeted drug delivery in conditions such as cancer or inflammation [107]. Temperature-responsive gels infused with essential oils could find applications in personal care products, where they release fragrances or therapeutic agents upon contact with the skin [108].

### 4.2. Stability of Bioactive Compounds

Many bioactive compounds in natural extracts, such as phenolics, flavonoids, and essential oils, are sensitive to environmental factors like light, heat, and oxygen. This sensitivity can lead to degradation during gel preparation, storage, and application, reducing their effectiveness [23,73]. For instance, curcumin and ascorbic acid are prone to oxidative degradation, which can compromise their antioxidant properties when incorporated into gels [109,110]. To overcome this limitation, encapsulation techniques, such as nanoencapsulation or microencapsulation, are used to protect sensitive bioactives from environmental stress [63]. Additionally, the use of stabilizers or synergistic combinations of bioactive compounds can enhance their stability within gel matrices [94].

Stabilizing bioactive compounds in gel formulations requires a multi-faceted approach, such as combining encapsulation technologies, optimized processing, and innovative solvents like natural deep eutectic systems (NADESs). NADESs, composed of natural metabolites, such as organic acids, sugars, and amino acids, enhance the solubility, bioavailability, and stability of bioactive compounds [111,112,113]. Their incorporation into gel systems not only improves functionality but also aligns with sustainable practices by offering eco-friendly and renewable alternatives to traditional solvents [112,113]. The unique properties of NADESs, including their ability to enhance solubility, reduce degradation, and provide a green chemistry solution, make them a promising addition to gel-based systems [102]. NADESs formulated with natural acids and sugars can solubilize flavonoids or polyphenols, allowing their stable integration into gel systems [114]. By integrating NADESs and other stabilization strategies, researchers can develop robust formulations that ensure the efficacy and longevity of bioactive compounds in diverse applications.

### 4.3. Interaction with Gel Matrices

The interactions between natural extracts and gel matrices play a critical role in determining the functionality and performance of the final product. However, these interactions can sometimes lead to undesirable effects, such as reduced gel strength, phase separation, or poor release profiles of bioactive compounds [16]. For instance, incorporating highly hydrophobic essential oils into hydrogels may result in an uneven distribution or aggregation, which negatively impacts the gel’s mechanical properties [16,90,115]. Similarly, phenolic compounds can interfere with crosslinking processes in certain gel systems, reducing structural integrity [52].

Optimizing the compatibility between natural extracts and gel matrices is essential and can be achieved through formulation adjustments, as well as the use of emulsifiers or surfactants [108]. Advances in the development of carriers for natural extracts have introduced systems such as NADESs, liposomes, nanoparticles, emulsions, and edible films, each demonstrating unique functionalities. However, each carrier comes with its own advantages and limitations, as summarized in Table 3.

Gels, particularly hydrogels and oleogels, are versatile carriers for bioactive compounds. Their high-water content supports hydrophilic extracts, while oleogels and nanoemulgels extend compatibility to hydrophobic compounds [5,37]. Gels also provide biocompatibility and sustained-release properties, making them suitable for applications in wound healing, food preservation, and cosmetics [103,104,105]. However, they face challenges, such as drying out over time and production complexities when modified for specific functionalities [116]. NADESs are gaining attention as eco-friendly carriers with high efficiency in extracting and stabilizing bioactive compounds [112,113]. They are formed from natural compounds, such as sugars, amino acids, and organic acids, creating non-volatile, biodegradable systems [111,112,113]. NADESs offer tunable solubility and stability, enabling the selective extraction of antioxidants and antimicrobials from natural sources with minimal waste. Furthermore, NADESs can complement gels by enhancing the solubility and bioavailability of poorly soluble compounds, expanding their application in pharmaceutical and cosmetic formulations [112,113]. However, more research is needed to address their scalability and long-term stability in complex systems. Liposomes and nanoparticles offer superior encapsulation efficiency and targeted delivery but require expensive and complex production processes [36,117]. Emulsions and nanoemulsions are cost-effective options for hydrophobic compounds but suffer from stability issues like phase separation [118]. Edible films, while eco-friendly and efficient in controlled release, lack the hydration capacity of gels [119].

### 4.4. Controlled Release and Bioavailability

Natural extracts significantly enhance the bioactivity of gels; however, achieving controlled release and high bioavailability of bioactive compounds remain a challenge. The release profile of these compounds depends on factors such as their solubility, interaction with the gel matrix, and environmental conditions [120]. Rapid release can result in a short duration of action, while overly slow release may lead to suboptimal bioactivity [115,121]. To address these issues, researchers are developing advanced gel systems, including hybrid gels and responsive hydrogels. These innovative systems enable tailored release profiles triggered by environmental factors, such as pH, temperature, or enzymes, thereby enhancing the therapeutic efficacy and functional performance of gels across various applications [122].

Nanotechnology offers tremendous potential for improving the performance of gels containing natural extracts. Nanoencapsulation techniques can enhance the stability, bioavailability, and targeted delivery of sensitive bioactive compounds, such as polyphenols and essential oils [115,123]. Additionally, nanoparticles integrated into gel systems introduce new functionalities. For instance, nanogels incorporating natural extracts and metallic nanoparticles demonstrate synergistic antimicrobial effects, making them highly suitable for biomedical applications [124]. In the food industry, nanocomposite-based gel systems can provide superior barrier properties, protecting food from microbial contamination and oxidative damage [8,125].

Emerging technologies like artificial intelligence (AI) and machine learning (ML) are accelerating innovation in gel research. These tools enable optimization of formulations by predicting interactions between natural extracts and gel matrices, determining ideal concentrations, and forecasting release profiles [126,127]. Furthermore, AI-driven approaches can analyze large datasets to identify promising natural extracts for specific applications, significantly reducing the time and cost of experimental screening. Advancements in 3D printing are also creating new opportunities for designing customizable gel systems. Printable hydrogel formulations containing natural extracts can now be used to fabricate complex structures tailored to specific needs [128]. In tissue engineering, 3D-printed gels with bioactive compounds can serve as scaffolds for organ or tissue regeneration, offering patient-specific solutions [9,116]. Similarly, in the food industry, printed gels have the potential to deliver precise doses of nutrients or flavors, paving the way for personalized nutrition.

### 4.5. Scaling Up Production and Addressing Knowledge Gaps

Scaling up the production of gels containing natural extracts presents a range of logistical challenges, particularly in the areas of sourcing, processing, manufacturing, and cost-effectiveness. The extraction and purification of bioactive compounds from natural sources are often time-consuming and resource-intensive, making large-scale production expensive and less sustainable [129,130]. Many natural extracts, especially those derived from rare or exotic plants, are available in limited quantities, leading to supply chain issues [131]. Seasonal variations, environmental factors, and overharvesting exacerbate these challenges, further complicating the consistent sourcing of raw materials [23,73].

The processes required to extract and purify these bioactive compounds are another major barrier. Conventional extraction methods may require large volumes of solvents and extensive processing, which are not only costly but also environmentally taxing [132,133]. Advances in extraction technologies, such as supercritical fluid extraction and green chemistry approaches, can help address these issues by improving yield and reducing environmental impact [32,133]. However, adapting these techniques for large-scale production remains a technical challenge. Additionally, ensuring uniform incorporation of natural extracts into gel matrices during large-scale manufacturing can be difficult [25]. Variability in the composition of natural extracts and differences in their physicochemical properties, such as solubility and viscosity, often results in inconsistent product quality [124,125].

Maintaining the stability of bioactive compounds is another critical issue. Many bioactive compounds are sensitive to environmental factors, such as temperature, light, and oxygen, and maintaining their stability throughout the production, storage, and distribution processes requires specialized formulations and packaging, which further complicates the logistics [97,98,99]. The scalability of production itself presents challenges. Traditional methods of gel production that work efficiently on a small scale often cannot be easily translated to industrial-scale operations [116]. Developing automated gel production techniques and continuous processing systems is essential to enhance scalability and cost-effectiveness, but these innovations require substantial investment in infrastructure and technology [9,116,126,127].

Finally, the cost of natural extracts and the processes involved in their incorporation into gels can be prohibitive, particularly for large-scale production [134]. High-value extracts from rare or exotic plants further increase expenses, making it difficult to balance cost with performance and market demands [32,125]. Efforts to reduce costs include utilizing by-products or waste materials from the food and agricultural industries as alternative sources of natural extracts [26]. For example, lemon balm and chokeberry pomace serve as cost-effective sources of phenolic compounds, offering both economic and environmental benefits [65]. Addressing these logistical challenges requires a combination of innovative technologies, such as automated production systems, and sustainable sourcing strategies to ensure the feasibility and scalability of gel production containing natural extracts [126,127,128].

While the integration of natural extracts into gel systems offers immense potential, there remain critical knowledge gaps that must be addressed for greater scientific and industrial relevance. Specifically, two key areas require deeper investigation: (1) Impact of natural extracts on gel structure: The incorporation of natural extracts into gel matrices may alter the physical and chemical properties of the gel. Bioactive compounds, such as phenolics and flavonoids, can interact with the gel’s polymer network through hydrogen bonding, ionic interactions, and hydrophobic forces [36,42,45,46,47]. These interactions may influence the gel’s rheological behavior, stability, and structural integrity. Further studies are needed to elucidate these mechanisms and optimize formulations for enhanced gel performance. (2) Influence of the gel matrix on bioactive interactions: Embedding natural extracts within a gel matrix may affect their bioavailability, stability, and interaction with biological targets [42,69,70,73,74,75,79,113]. The gel environment could modulate the release kinetics of active compounds, thereby altering their efficacy in therapeutic or antimicrobial applications [22,39,43,79,109,122]. Understanding how gel matrices influence the accessibility and reactivity of these bioactives is crucial to advancing their functionality [69,70,88,92,95,135]. Addressing these knowledge gaps will enhance the relevance and applicability of natural extract-enriched gels. By employing advanced analytical techniques, such as molecular modeling and spectroscopy, researchers can gain insights into these complex interactions [101,136]. Additionally, tailoring gel systems to optimize the compatibility and performance of natural extracts will enable the development of multifunctional materials that meet the demands of diverse industries [16,42,88,89,92,126,135,137].

### 4.6. Regulatory and Safety Concerns

The incorporation of natural extracts into gel systems for food, pharmaceutical, and cosmetic applications faces stringent regulatory and safety requirements. Ensuring the safety, efficacy, and regulatory compliance of these gels poses a significant challenge. Certain essential oils and bioactive compounds, particularly at high concentrations, may exhibit toxicity or cause adverse effects, necessitating comprehensive toxicological evaluations [86,96]. Regulatory bodies, such as the Food and Drug Administration (FDA) and European Food Safety Agency (EFSA), require adherence to established guidelines, including rigorous safety testing and efficacy validation, to ensure that natural extract-based gels meet the required standards [32,138]. Transparency in labeling, particularly regarding the source and composition of natural extracts, is critical to gaining consumer trust and facilitating market acceptance [139,140].

In addition to safety concerns, growing environmental awareness is driving the demand for sustainable and biodegradable gel systems [139]. Natural polymers, such as alginate, chitosan, and cellulose, when combined with plant-based extracts, are being explored as eco-friendly alternatives to synthetic materials [122,134]. These materials not only address consumer preferences for environmentally conscious products but also reduce the ecological footprint of gel production [73,88]. Moreover, utilizing agricultural by-products, such as fruit peels or seed husks, as sources of both natural extracts and gel-forming materials offers a promising solution for achieving sustainability [65]. This zero-waste approach aligns with circular economy principles, reduces costs, and minimizes resource wastage [26,65].

Future advancements in this field will depend on the harmonization of regulatory standards across regions. Unified guidelines for the safety, efficacy, and labeling of natural extract-based gels will simplify compliance and foster greater market acceptance [139,140]. Raising consumer awareness through education and targeted marketing campaigns is equally important. Highlighting the sustainability, safety, and effectiveness of natural extract-based gel systems can drive demand and encourage widespread adoption [32,138]. As research progresses, the integration of sustainable practices and adherence to clear regulatory frameworks will be pivotal in the development and commercialization of these innovative systems.

### 4.7. Sustainability and Green Chemistry in Gel Production

Sustainability and green chemistry are becoming integral to the design and development of gel systems incorporating natural extracts, as industries shift toward eco-friendly and environmentally responsible solutions [111]. A key area of focus is the adoption of biodegradable and renewable polymers, such as alginate, cellulose, chitosan, and starch, which not only reduce environmental impact but also maintain the structural and functional integrity of gels [112,113,141]. These materials present an eco-friendly alternative to synthetic polymers, which are often derived from non-renewable petroleum sources and pose challenges related to waste accumulation and degradation [26]. By utilizing biodegradable polymers, gel systems can contribute to reduced pollution and align with growing consumer demands for sustainable products [141,142]. In addition to material selection, greener extraction methods are gaining prominence. Among these, ionic liquids have emerged as innovative and efficient extraction media, providing an environmentally friendly alternative to traditional organic solvents [32,133]. Ionic liquids offer tunable properties, such as low volatility, high thermal stability, and selective solubility, making them ideal for the sustainable extraction of bioactive compounds from natural sources [143]. These properties minimize chemical waste and reduce the environmental footprint of extraction processes, supporting the principles of green chemistry [26]. Furthermore, integrating supercritical fluid extraction and microwave-assisted extraction techniques allows for energy-efficient and solvent-free processing, enhancing the overall sustainability of the production pipeline [132,133]. The use of renewable resources is another cornerstone of sustainable gel production. By replacing synthetic solvents with bio-based or recyclable alternatives, industries can decrease their dependence on fossil fuels and mitigate the environmental consequences of solvent disposal [87,111,114,144]. Optimizing energy-efficient processes, such as continuous-flow manufacturing systems, further reduces carbon emissions and operational costs. These efforts not only enhance the sustainability of gel production but also improve the economic feasibility of incorporating natural extracts into large-scale applications [9,116,126,127].

Beyond production methods, the concept of a circular economy is being embraced in gel development. Agricultural by-products, such as fruit peels, seed husks, and other food processing residues, are being utilized as sources of both natural extracts and gel-forming materials [26,65]. This zero-waste approach leverages abundant and underutilized biomass, transforming it into valuable inputs for therapeutic, cosmetic, and even food-related applications [26]. By reducing resource waste and promoting the reuse of materials, such strategies contribute to a more sustainable production cycle. These advancements in sustainability and green chemistry not only address environmental preservation goals but also enhance the therapeutic and cosmetic potential of gel systems [74,87,111,129,145]. As research progresses, further innovations in renewable materials, efficient extraction technologies, and energy optimization are expected to expand the possibilities for sustainable gel production [26,65]. By aligning with global environmental goals, industries can deliver high-performance, eco-friendly gel systems that meet consumer demands while fostering environmental stewardship.

### 4.8. Future Perspectives

Overcoming these challenges requires a multidisciplinary approach involving chemistry, materials science, biotechnology, and engineering. Future research should focus on the following:

- Developing more efficient and sustainable extraction methods for natural bioactives.
- Investigating the molecular interactions between natural extracts and gel matrices to optimize their compatibility and performance.
- Designing advanced gel systems with responsive or multifunctional properties for targeted applications.
- Exploring the use of alternative, low-cost natural sources to reduce production costs.

The integration of nanotechnology, machine learning, and high-throughput screening methods can further accelerate the development and optimization of natural extract-based gels, paving the way for innovative solutions in the healthcare, food, and environmental sectors [127,130]. The future of gels with natural extracts lies in interdisciplinary collaboration. Integrating expertise from chemistry, biology, materials science, and engineering will enable the development of next-generation gel systems [116,128]. Partnerships between academia, industry, and regulatory bodies can further ensure that research translates into practical applications.

## 5. Conclusions

The integration of natural extracts into gel systems offers transformative potential across industries, including pharmaceuticals, food technology, and cosmetics. These gels provide enhanced antioxidant and antimicrobial properties, driven by bioactive compounds, such as polyphenols, flavonoids, and essential oils. The synergy between gel matrices and natural extracts enables the development of multifunctional materials with applications in wound healing, controlled drug delivery, food preservation, and cosmetic formulations. Moreover, the focus on sustainability and green chemistry has paved the way for eco-friendly innovations, incorporating biodegradable polymers and agricultural by-products while employing advanced extraction methods like supercritical fluids and ionic liquids.

## 6. Method

This narrative review was conducted in three steps—performing the search, reviewing abstracts and full-texts, and discussing the results—following the procedure outlined by Lins et al. [26]. PubMed, Scopus, ScienceDirect, and Google Scholar databases were searched to identify relevant studies for the review. The keyword “natural extract” was combined with terms such as gels, gelation, encapsulation, hydrogels, gel-based, coating, oleogels, edible films, natural deep eutectic systems, antioxidant, and antimicrobial. After completing the search, the abstracts were screened to ensure they addressed the topic of interest. Duplicate entries were removed, and the abstracts of the remaining articles were reviewed to confirm they met the inclusion criteria for the review.

## Figures and Tables

**Table 1:** The interactions of natural extracts with various gel-based formulations as antioxidant agents.

Extracted Sources	Active Compounds	Gel Forms	Effect of Adding a Natural Extract	References
Tea polyphenols (TPs)	Polyphenols	Oleogel	Oleogel with TP showed an oxidative stability value of 31.31%, compared to 68.84% in those without TP, indicating better resistance to oxidation during storage.	[17]
*Syzygium aromaticum* Essential oil	Eugenol, terpenes and terpenoids, sesquiterpenes, phenolic compounds, and aldehydes	Hydrogel	Hydrogel with essential oil exhibited a slight scavenging potential towards the DPPH radical, calculated at less than 10%.	[18]
Rosemary (*Rosmarinus officinalis*) essential oil	Flavone compounds, diterpenes, steroidal derivatives, and triterpenes	Nanoemulgel	Nanoemulgel exhibited strong antioxidant activity, with a value of 22.38 ± 0.7 μg/mL, compared to Trolox (2.7 ± 0.5 μg/mL).	[19]
*Kunzea ericoides* leaf	Alkaloids, organic acids, amino acids, polyphenols, aromatic acids, depsides, flavonoids, dihydroxy coumarin, and triterpenoids	Hydrogel	The hydrogel with *Kunzea ericoides* leaf extracts showed a significantly higher ABTS inhibition rate (35.35 ± 1.58%) compared to the gelatin methacryloyl hydrogel (6.60 ± 0.50%) and demonstrated an excellent DPPH inhibition rate of 28.43 ± 1.05%, while the gelatin methacryloyl hydrogel exhibited no activity.	[61]
*Aloe vera* leaf	Phenylpropanoids and coumarins, flavonoids, phenylpyrone and phenol derivatives, and phytosterol	Edible film	The incorporation of aloe vera extracts into the chitosan film showed no significant difference in DPPH scavenging activity but demonstrated higher ABTS scavenging activity (26.1%) compared to chitosan alone (22.7%).	[62]
*Moringa oleifera* seed	Polyphenols, flavonoids	Encapsulation	The extract and encapsulated product showed initial DPPH inhibitions of 38.67% and 31.29%, respectively, but the encapsulated product maintained 14.25% inhibition by the fourth week, while the extract dropped to 3.79%. ABTS inhibition was higher, with values of 42.34% and 36.78%, reflecting effective antioxidant interactions in *Moringa oleifera* seeds.	[63]
Blackcurrant pomace extract	Phenolics, anthocyanins	Emulsion gel	Emulsion gel with blackcurrant pomace extract increased the oxidative stability of Vienna sausages during storage.	[64]
Waste of lemon balm (LB) and chokeberry pomace (ChP)	Terpenoids, flavonoids, phenolic acids, tannins, and essential oils	Alginate Film	Films with LB showed QUENCHER_DPPH_ = 469.0–1174.9 μmol TE/100 g and QUENCHER_CUPRAC_ = 162.6–377.1 μmol TE/100 g) more than films with ChP (QUENCHER_DPPH_ = 348.3–819.0 μmol TE/100 g and QUENCHER_CUPRAC_ = 33.7–252.1 μmol TE/100 g)	[65]
*Oliveria decumbens* Vent. essential oil	Phenolics, essential oils	Gelatin-based films	Increasing the *Oliveria decumbens* Vent. essential oil concentration from 3% to 9% significantly enhanced antioxidant activity. At 9%, the highest DPPH inhibition was 85.89%, with a FRAP value of 0.66 mg/mL.	[66]
Tragacanth gum and clove	Phenolics, polysaccharides, eugenol, gallic acid, flavonoids, and anthocyanins	Hydrogel	The hydrogel containing tragacanth gum and 5% clove extract exhibited DPPH inhibition of up to 92%, outperforming tragacanth gum or clove extract alone.	[67]
Egg white protein + apple polyphenol	Polyphenols, protein	Oleogel	The crosslinking of egg white protein and apple polyphenol in the oleogel inhibited the generation of oxidation products by 65%.	[68]
Ovalbumin amyloid fibrils + tannic acid (TA).	Phenolics, protein	Hydrogel	The hydrogel containing 2% TA exhibited the highest FRAP value of 1.65 mM. In comparison, hydrogels with 1% TA and 0.5% TA showed lower FRAP values of 1.07 mM and 0.66 mM, respectively. The 2% TA hydrogel also displayed significant DPPH activity of 85.99%.	[69]
Ovalbumin amyloid fibrils + epigallocatechin gallate (EGCG)	Polyphenols, protein	Hydrogel	The hydrogel containing ovalbumin amyloid fibrils and EGCG exhibited antioxidant activity due to the presence of EGCG. With a 2% EGCG concentration, the hydrogels achieved maximum DPPH radical scavenging activity of 86.92% and FRAP capacity of 2.99 mM.	[70]
*Aloe vera* gel	Flavonoids, phenolics	Chitosan nanoparticles	Incorporated *A. vera* gel in nanoparticles exhibited an IC_50_ of 81.7 μg/mL for DPPH scavenging, which was reduced by 40% when *A. vera* gel was used alone.	[71]
Apple extract	Flavonols, flavan-3-ols, phenolics	Oleogel	Oleogel-released complex of phenolic compounds penetrating into the epidermis showed the strongest DPPH free radical scavenging activity (281.8 ± 14.1 µM TE/L).	[72]
Beeswax	Wax esters, free fatty acids, and alcohols	Oleogel	Replacing beeswax with its fractions as a gelator can result in oleogels with enhanced oxidative stability.	[73]
Green tea extract	Polyphenols	Oleogel	Green tea extract oleogel reduced the levels of free radicals and lipid hydroperoxides.	[74]
Beeswax, sunflower wax, and carnauba wax	Phospholipids, glycolipids, free fatty acids, transition metals, waxes, phenolic compounds	Oleogel	The addition of beeswax, sunflower wax, and carnauba wax protected the oxidation of thymoquinone, the main phenolic compound in black cumin oil oleogel.	[75]
Birch outer bark extract	Betulin, betulinic acid, and lupeol	Oleogel	Polyphenols in birch outer bark extract provide antioxidant properties with a protection factor of 3.2, which is equal to or even better than that of synthetic compounds.	[76]
Rosemary extract	Phenolic compounds	Hydrogel, Oleogel	Coating sardine fillets with hydrogel and oleogel combined with rosemary extract delayed the oxidation process and reduced oxidation values by 75%.	[77]
*Centella asiatica*	Aromatic ethers, trans alkenes, alkanes, amines, carboxylic acids, nitrocompounds, nitrites, esters, acetates, alcohols, ketones, esters, cisalkenes	Bigels	The addition of *C. asiatica* extract to the bigels enhanced their free radical scavenging ability by 20%.	[78]
Soybean lipophilic protein + thyme essential oils + glycerol monolaurate (GML)	Phenolic compounds, protein	Oleogel	Oleogel with 2% GML exhibited the highest DPPH radical scavenging rate of 94.7% ± 3.56, while its ABTS scavenging rate was 85.77% ± 3.38.	[79]
*Amomum villosum* Lour. extract	Flavonoids and polyphenols	Oleogel	The addition of *Amomum villosum* Lour. extract delayed the oxidation of the oil in the oleogel by 33% compared to the sample without the extract.	[80]
*Calendula officinalis* extract	Phenolic acids and flavonoid compounds	Hydrogel	*C. officinalis*-loaded hydrogel significantly increased DPPH scavenging activity from 50% to 70% by increasing the concentration from 1.3 to 4.5 mg/L.	[81]
Olive leaf	Phenolic compounds	Edible film	The increase in olive leaf extract concentration resulted in an enhanced DPPH scavenging activity of the film.	[82]
Keratin from chicken feathers	Keratin, citric acid	Film	The keratin/citric acid-modified cellulose nanocrystals film achieved 50% DPPH scavenging activity within 3 h.	[83]

**Table 3:** Advantages and disadvantages and applications of gels compared to other carriers.

Carrier Type	Advantages	Drawbacks	Examples of Applications
Gels	- High water content supports hydrophilic compounds.	- Limited compatibility with hydrophobic compounds unless modified.	- Wound healing, food packaging, cosmetics
	- Versatile (hydrogels, oleogels, nanoemulgels)	- Production requires specific conditions for structural integrity.	- Delivery of antioxidants and antimicrobials
	- Biocompatible and non-toxic.	- Susceptible to drying out, reducing stability.	
Natural Deep Eutectic Systems (NADESs)	- Eco-friendly and biodegradable.	- Limited studies on long-term stability in complex formulations.	- Extraction of bioactive compounds, cosmetic and pharmaceutical formulations
	- Highly effective in extracting and stabilizing bioactive compounds.	- Compatibility with large-scale production needs further exploration.	
	- Minimal chemical waste due to low volatility and tunable solubility.		
Liposomes	- Excellent encapsulation for both hydrophilic and hydrophobic compounds.	- Expensive to produce and stabilize.	- Drug delivery, cosmetic formulations
	- Protects bioactive compounds from degradation.	- Sensitive to environmental factors (e.g., temperature, pH).	
Nanoparticles	- High surface area allows efficient encapsulation and targeted delivery.	- Synthesis can be complex and costly.	- Targeted drug delivery, bioactive stabilization
	- Can deliver bioactives to specific sites.	- May pose environmental or biological risks at higher concentrations.	
Emulsions	- Simple and cost-effective to produce.	- Stability issues like phase separation over time.	- Food preservation, cosmetic formulations
	- Easily incorporates hydrophobic compounds.	- Lack of structural integrity for some applications compared to gels.	
Edible Films/Coatings	- Provide controlled release of bioactives and are eco-friendly.	- Limited mechanical flexibility compared to hydrogels.	- Food packaging, antioxidant, and antimicrobial delivery
Nanoemulsions	- Excellent for hydrophobic compounds due to nano-sized droplets.	- Require high energy input or specialized stabilizers.	- Delivery of essential oils and natural extracts in food and cosmetics

## Data Availability

Not applicable.
